# Supplementary material for: Are carbon emissions trading and green financial instruments synergistic? -Comprehensive quantitative research based on content analysis
Source: PLoS One. 2024 Mar 7;19(3):e0298601. doi: 10.1371/journal.pone.0298601 (PMC10919601; doi:10.1371/journal.pone.0298601)
Supplement: S1 Data — (ZIP) [file pone.0298601.s001.zip › Data/地级市Z_F工作报告.zip!/╜¡╬≈/╨┬╙α╩╨/└┤╫╘╣1⁄2╓┌║┼í╛┬φ┐╦╩2╛▌═°í┐_2019.docx]

各位代表：

现在，我代表市人民政府向大会报告政府工作，请予审议，并请各位市政协委员和列席会议的同志提出意见。

一、2018年工作回顾

2018年，在省委、省政府和市委的坚强领导下，全市上下深入学习贯彻习近平新时代中国特色社会主义思想和党的十九大精神，坚持稳中求进工作总基调，贯彻新发展理念，落实高质量发展要求，一心一意谋发展，一以贯之抓落实，务实苦干，砥砺前行，在高质量跨越式发展的道路上迈出了坚实步伐。预计（下同）全年实现生产总值1100亿元，增长8.3%；固定资产投资增长10.6%；社会消费品零售总额299.2亿元，增长11%；实际利用外资4.6亿美元、增长6%，省外2000万元以上项目资金505.5亿元、增长7%；城镇居民人均可支配收入37557元、增长8%，农村居民人均可支配收入17990元、增长8.5%；居民消费价格指数涨幅控制在3%以内。需要向各位代表报告的是，2018年我们全力提高财政收入质量，坚决落实各项减税降费政策，下决心挤压多年来积攒的水分，全市财政总收入144.25亿元，税收占比达89.92%、列全省第一，提高15个百分点，以“短痛”换得了“长利”。

（一）全力以赴强产业，发展质量持续向好。三次产业结构调整为5.1∶51.1∶43.8。工业经济提质发展。规模以上工业增加值增长8.3%，主营业务收入增长8%。全面落实支持新钢跨越发展30条措施，钢铁产业主营业务收入增长9.7%，新钢联天钢构、优特钢带加工配送中心和中冶新材料14万吨新能源汽车用电工钢等一批产业延伸合作项目，加快实施或建成投产，新钢带动作用更加彰显；渝水区获评全省钢铁产业优化升级专项试点示范区。出台实施支持锂电产业发展30条措施，产业集群三年倍增计划顺利完成，新能源、光电信息、装备制造产业增加值分别增长9.6%、11.3%和14.7%；高新区列为科技部动力电池创新型产业集群试点。新兴产业增加值占规模以上工业比重达40.7%，规模以上工业实现利润较快增长，工业发展的质量和效益明显提升。高新区在全国国家级高新区的综合排名晋升4位、列第94位，工业主战场地位更加凸显。扎实开展企业精准帮扶，深入落实降成本优环境专项行动，以“真心”为企业节约“真金”32亿元。引进韩国SNM公司和赣商科技集团成功重整赛维。新增入规工业企业45户。第三产业加快发展。实现增加值483亿元、增长9%，新增入规服务业企业16户。出台《防控金融风险支持实体经济发展的若干意见》等系列政策，与银行同频共振优化金融环境，全市金融机构人民币存、贷款余额分别增长9.2%和7.2%，不良贷款率下降至4.15%，彻底扭转了长期全省垫底的不利局面。出台实施企业上市“三年行动”计划，沃格光电在上交所成功上市，成为全省“映山红行动”第一股；赣锋锂业在港交所成功上市，成为全省第二家“A+H”股上市企业。陆路口岸查验区建设有序推进，新余至宁波舟山港“五定”班列顺利开通。太平洋购物广场、喜盈门•范城商业综合体和暨阳上亿广场相继建成营业。旅游“双创”工作相互促进，我市被评为全国百佳乡村旅游目的地，彩色村被评为中国美丽休闲乡村；成功引进省旅游集团投资仙女湖旅游，央视七夕晚会和中国诗词大会七夕特别节目在我市成功录制，仙女湖旅游影响力与日俱增，全市接待游客量和旅游总收入均增长20%以上。第三届“天工杯”工业设计大赛、第四届南方农业（工程）机械展销会和第五届麻博会成功举办。现代农业稳步发展。建成高标准农田6.2万亩，52家省级以上龙头企业实现销售收入100.1亿元。农村土地确权颁证、农村集体资产清产核资工作全面完成，农村土地经营权流转率达54.2%。特色农业势头良好，新余蜜桔、高产油茶等产销两旺。循环农业加快壮大，沼气发电项目年处理农业废弃物达40万吨，蘑菇产业年处理农业废弃物达10万吨。科技创新助推发展。国家创新型试点城市创建稳步推进，科技进步对经济增长贡献率达57%。全社会研发经费投入占生产总值比重达1.53%。新增国家级企业技术中心1家、省级研发平台3家、国家高新技术企业30家，获批省“海智计划”工作站1家，新增院士工作站2家。全面落实人才政策30条，新增国家“万人计划”1人、省“双千计划”5人、省百千万人才工程3人、省主要学科学术和技术带头人4人，获省政府特殊津贴3人。6项科技创新成果获全省科技奖励大会表彰，获奖数量与南昌市并列全省第一；专利申请量2700件、授权量922件，每万人有效发明专利拥有量达3.12件，人才创新创造的活力不断喷涌。

（二）聚精会神抓项目，发展后劲不断增强。项目建设再创新佳绩。坚持“项目为王、项目立命”理念，全年共安排重点建设项目223个，完成投资235亿元、增长18%，其中工业项目个数及投资额占比均超过50%。消化批而未用土地12692亩，有力保障了项目建设用地需求。一批打基础、利长远的基础设施项目加速推进，环城路路基、桥梁、通涵工程均完成75%以上，省道S222凤阳至山塘下改建工程进展顺利；城乡供水一体化基本实现“50户以上自然村供水管网全覆盖、具备通水条件的自然村当期开户率50%、水质合格率100%”三大目标，城市供水应急备用水源狮子口水库全面建成；蒙华铁路（新余段）和国电投分宜电厂扩建加快推进，新余煤储基地项目顺利开工，大唐新余二期异地扩建项目前期工作取得实质性进展，赣西能源中心呼之欲出；全国铁路二级物流基地项目成功列入中国铁总2019-2020年规划；江西（赣西）军民融合军粮综合保障配送基地成功落户我市，赣江货运码头建设合作协议正式签订；大岗山水库建设、江口水库水位提升工程、新钢公司研发基地和生活服务基地向仙女湖区转移项目前期工作启动；探获渝水区石竹山硅灰石矿石量达1.1亿吨，储量世界第一。一批调结构、促转型的工业项目落地实施，赣锋锂业2万吨电池级氢氧化锂、江西雅保2.5万吨氢氧化锂、科能4亿安时锂电池等60个重点工业项目建成投产；新钢百亿产业转型升级改造全面开工；建筑科技产业园、装配式建筑产业园、高新区数控机床产业园等特色产业园加速推进。一批补短板、暖民心的民生项目有序推进，市妇幼保健院扩建、市中医院迁建和袁河医院新建项目开工，袁河公园、湿地公园三期、北湖公园东大门、赣西大道和白竹路“白改黑”等项目顺利建成，5条背街小巷、2个老旧小区基础设施改造全面完成。项目建设所积蓄的澎湃动能，正在拉动实体经济稳步复苏，推动社会事业全面进步。招商引资实现新突破。主攻产业招商、狠抓专题招商、致力招大引强，举办或参加各类招商活动32场，共引进2000万元以上项目176个、合同金额527.4亿元，其中北京升华15000台电梯、信诚电子陶瓷基板及元器件、分宜双胞胎生态化养殖等投资10亿元以上项目18个、总投资310亿元。“三争”工作闯出新路径。科学编制“三争”任务表，梳理各类信息936条，共争取上级财政资金62.3亿元、增长10%，其中保障性安居工程建设获上级补助资金5.2亿元，资源枯竭型城市获中央、省资金2.86亿元。

（三）攻坚克难促改革，体制机制逐步理顺。60项重点改革项目年度任务全面完成。行政管理体制改革方面。在全省率先推行“一窗式”综合服务平台试点，市级政务服务事项全部纳入市行政服务中心集中办理。865项市本级政务服务事项实现“一次不跑”或“只跑一次”；不动产登记改革全省领先，中央电视台进行了专题报道，14项特色服务得到自然资源部肯定和推广；商事制度改革深入推进，企业注册开办压缩至5个工作日；“证照合一”改革走在全省前列，办理速度提高80%以上；共取消调整各类证明事项255项，企业和群众办事更加方便快捷，被列为全省“放管服”改革工作试点市。升级版“12345”政府服务热线成功上线运行，企业和群众诉求渠道更加畅通。机构改革稳步推进，分宜县融媒体改革入选全国改革开放40周年地方改革创新40案例。高铁新区正县级管委会成功获批，利用亚洲开发银行贷款项目进入实施阶段，为主城区扩容提质拓展了新的发展空间。经济管理体制改革方面。新宜吉转型合作示范区成功举办合作大会，赣粤高速南安收费站拓宽改造等15个项目陆续完工；成功举办联合招商推介会，签约项目18个、总投资达118.9亿元。积极化解政府性债务，98%以上的存量债务转化为低息政府债券，债务结构不断优化。国有资产盘活整合工作成效明显，市属国有企业实现营业收入增长70.9%。开发区改革扎实推进，向高新区下放权限184项，明确了各开发区首位产业和主攻产业。社会管理体制改革方面。政府定价内容减少26%，车用天然气基准销售价全面放开，仙女湖景区门票价格下调16.7%。社会信用体系建设深入推进，管理体制进一步理顺。罗坊智慧小镇成为全省首个乡镇大数据综合服务平台，得到刘奇书记肯定；分宜锻压厂社区工作法入选全国百个优秀社区工作法。乡镇商会建设经验全省推广。完成市智慧养老服务指挥中心和25个社区居家养老服务中心建设，成功入选第三批中央财政支持开展居家和社区养老服务改革试点城市。殡葬改革稳步推进，51个农村公益性墓地、新余人文纪念园一期和分宜县殡仪馆一期工程建设开工。

（四）不遗余力抓环保，生态环境日益改善。环境治理扎实有效。全力打造“保家行动”升级版，可限养区924家畜禽养殖场已关停拆除或生态化改造；新铺设园区污水管网66.6公里，工业污水处理厂日均处理量实现翻番。建成村镇生活污水处理设施38座、配套管网140公里，58项流域截污治污体系建设等水污染治理工程已完成53项。全面开展“清河行动”，扎实推进入河湖排污口排查整治，全市入河湖支流水质达标率达79%，完成省政府下达的消灭劣Ⅴ类水目标任务。深入推进“蓝天行动”，新钢6米焦炉烟气脱硫脱硝等项目建成投用，建成进城货运车辆强制免费冲洗平台6个，绿化硬化裸露土105万平方米，非煤矿山综合整治稳步推进，主城区烟花爆竹禁燃禁放成效显著，圆满完成省政府下达的PM2.5年度目标任务，城区空气质量优良率达91.5%。重点行业企业用地土壤污染状况调查全面启动。扎实推进环保督察发现问题整改，科学论证影响仙女湖水质的污染源构成，得到中央环保督察组和省委省政府的理解认可，从而推动袁河全流域联合治理。大力开展环保专项执法行动，立案查处环境违法行为190件。608个新农村建设点年度任务全面完成，分宜小康用电示范县建设工程顺利完工，建成农村公厕2058座；扎实开展城乡环境综合整治，成功通过农村生活垃圾治理国检验收。生态制度不断健全。全面推行林长制，完善落实河长制湖长制，编制《仙女湖流域（新余段）水环境综合治理总体方案（2018-2020年）》，完成孔目江饮用水水源地保护区划分调整。《仙女湖水体保护条例》《新余市畜禽养殖污染防治条例》《江西仰天岗国家森林公园保护条例》相继实施，环保制度体系更加健全。我们相信，通过全市上下的不懈努力，一定能实现“蓝天常在、碧水长流、空气常新、美丽与发展同行”。

（五）矢志不渝谋福祉，民生保障持续进步。年初确定的40件民生实事中，除两个项目因群众有不同意见暂缓实施外，其余38件实事全部完成。脱贫攻坚扎实推进。紧紧围绕精准这个核心，新识别贫困户504户1017人，建成光伏扶贫联村电站4个、村级光伏扶贫电站140个、就业扶贫车间75个，实现2620名贫困人口稳定脱贫、9个省级贫困村摘帽。扎实开展“播爱使者”行动，306对农村大龄贫困人员成功“脱单”。城镇贫困群众脱贫解困工作全面启动，完成特困人员、低保对象、支出型贫困低收入家庭对象认定。就业创业成效明显。新增城镇就业2万人，新增转移农村劳动力1.6万人，获批省级创业孵化示范基地2个。新增发放创业担保贷款3.7亿元，扶持2429人次创业，带动11913人次就业。社会保障更加健全。发放全国试点地区首张电子社保卡。医保制度改革深入推进，按病种分值付费为主的多元复合式支付方式全面推行，新增3家跨省异地就医定点医院。城乡低保人均月补差分别达380元和255元；农村特困人员集中、分散供养月标准分别提高至455元和350元，城镇特困人员供养月标准提高至755元，困难群众生活得到改善。“颐养之家”建设基本实现农村符合条件有需求的老人全覆盖，人民群众表示“蛮恰噶”。已拆“三房”846.1万平方米，已建“三园”111.3万平方米，发放廉租住房租赁补贴3186户，改造垦区危旧房6120户、棚户区6123户。社会事业持续进步。新建成高新四小、分宜四小2所学校，渝水七小完成主体工程，市九中、渝水六小、城北幼儿园新建教学楼投入使用，新增学位7220个。出台支持新余学院发展30条措施；校企、校地合作机制不断完善，6所职业院校与30家企业开展现代学徒制合作。国家文化消费试点城市创建工作获国家文化和旅游部通报奖励，第四届中国（新余）傅抱石文化艺术节、新余2018中华全国爱情集邮文化嘉年华活动成功举办。医疗卫生体制改革不断深化，在全省率先建成医学影像、医学检验、医学病理等3个第三方医学共享中心，家庭医生签约补助标准提高至人均48元，签约覆盖率稳步提升。成功举办环鄱阳湖国际自行车大赛、环仙女湖国际马拉松等赛事。第十五届省运会上获金牌数列全省第四。社会治理能力显著增强。坚持“先立规矩后办事”，房地产开发领域历史遗留问题得到逐步化解，已为13个小区的3347套房产办理了不动产登记。全国文明城市、国家卫生城市和国家食品安全示范城市“三城”同创扎实推进。成立12个安全生产专业委员会，建立安全生产隐患与问题强力整治机制，扎实开展十大专项整治行动，没有发生重大安全事故。法治新余和平安新余建设深入推进，国家安全人民防线进一步加强，扫黑除恶专项斗争打掉一批涉黑涉恶团伙，公众安全感满意度排名全省第一，人民生活殷实安康。统计、海关、民族宗教、外事侨务、涉台事务、档案、地方志、公积金、机关事务、防灾减灾、妇女儿童、青少年、老龄、科普、气象、人防、电力、邮政通讯、烟草、盐业、海事、民兵预备役、社科研究、红十字会人道援助和慈善、残疾人事业等工作取得新成绩。

（六）持之以恒转作风，“五型”政府建设成效明显。工作作风持续好转。扎实推进省委巡视发现问题整改，全力开展“转变作风、坐实结果”活动和“五型”政府建设，坚决整治政府系统“怕、慢、假、庸、散”等作风顽疾，干部作风向“勇、快、实、进、聚”加快转变。履职能力不断提升。全面推进依法行政，严格落实法治政府建设和民主集中制，行政决策科学化、民主化、法治化水平稳步提升。健全正向激励和容错纠错机制，大力倡导“事事马上办、人人钉钉子、个个敢担当”的精神，形成了对标先进、比学赶超、争创一流的干事氛围。防腐堤坝加快构筑。严格落实党风廉政建设主体责任和“一岗双责”，坚决落实中央八项规定，重拳整治侵害群众利益的不正之风，坚决反对特权思想、特权现象，全市政府系统呈现出风清气正、人心思进的良好局面。

各位代表！过去的一年，政府工作有了新的进步。这得益于省委省政府和市委的坚强领导，得益于市人大的法律监督和市政协的民主监督，得益于社会各界和全市广大人民群众的积极作为。在此，我代表市人民政府，向全市广大工人、农民、知识分子、干部和历任老领导、老同志，向各民主党派、工商联、无党派和社会各界人士，向驻市人民解放军指战员、武警官兵、公安干警和消防救援队伍指战员，向驻市中央和省属单位，致以崇高的敬意！向所有关心、支持新余发展的同志们、朋友们，表示衷心的感谢！

回顾过去一年的工作，我们清醒地看到，全市经济社会发展仍面临不少困难和问题。产业发展方面：经济总量依然偏小，产业聚而不强，大部分企业缺乏核心竞争力，抵御市场风险的能力较弱；产业结构不尽合理，新兴产业受科技、人才等因素限制，成长壮大之路艰巨而漫长，依靠几个重点企业支撑发展的状况仍未得到根本改观；前些年个别企业对金融环境造成的影响仍未消除，修复金融生态、重塑金融机构对新余发展的信心、疏通中小企业发展的金融血脉还有很长的路要走。民生改善方面：经济增长下行压力与保民生、保运转、保生态等刚性支出增长并存，加之政府债务管控政策趋严，原有债务还本付息高峰期的到来，很多弥补城市基础设施建设短板、提升公共服务供给水平等想做的事只能分步推行；工业园区布局不尽合理，环保设施建设欠账较多，整治突出环境问题代价巨大、修复生态系统过程漫长，特别是仙女湖水质受上游来水影响较大，整治效果还不理想；城区中小学“大校额、大班额”问题依然突出，孩子们还上不好学；优质医疗资源总量相对不足、分布不均，群众意见还比较大，要满足人民日益增长的美好生活需要，还有许多工作要做。社会治理方面：工程建设、社会保障等领域积压多年的历史遗留问题还没有得到根本解决，群众还不满意、企业还不乐意，维护社会稳定任务更为艰巨繁重；少数干部服务意识和法治观念不强、工作作风不实、担当精神不够，形式主义、官僚主义不同程度存在，一些领域不正之风和腐败问题仍时有发生，转变政府职能、锻造“五型”干部队伍依然任重道远，等等。上述困难和问题，有些我们已经找到了破解办法，正在逐步化解；有些还在深挖问题根源，努力探索解决之方。冰冻三尺非一日之寒，融化坚冰也非一日之功，市人民政府一定勇挑时代赋予的重任，尽心竭力做好工作，使人民政府不辜负人民重托。

二、2019年工作安排

2019年，是新中国成立70周年，是决胜全面建成小康社会第一个百年奋斗目标的关键之年。我们将坚持以习近平新时代中国特色社会主义思想为指导，深入贯彻落实党的十九大及十九届二中、三中全会和中央经济工作会议精神，围绕从更高层次贯彻落实习近平总书记对江西工作的重要要求，按照省委“二十四字”方针，认真落实省委十四届七次全会和市委八届七次全会决策部署，坚持稳中求进工作总基调，坚持新发展理念，坚持推进高质量发展，坚持以供给侧结构性改革为主线，坚持深化市场化改革、扩大高水平开放，继续打好三大攻坚战，统筹推进稳增长、促改革、调结构、惠民生、防风险各项工作，促进经济社会持续健康发展，为共绘新时代江西物华天宝、人杰地灵新画卷贡献新余力量。

全市经济社会发展的主要预期目标是：生产总值增长8.2%左右；财政总收入增长5%左右；规模以上工业增加值增长8.2%左右，主营业务收入增长8%左右；固定资产投资增长9%左右；社会消费品零售总额增长10%左右；外贸出口稳中提质；实际利用外资增长6%左右，省外2000万元以上项目资金增长7%左右；城镇居民人均可支配收入增长8%左右，农村居民人均可支配收入增长8.5%左右；居民消费价格指数涨幅控制在3%左右。节能减排完成省下达的计划任务。

围绕上述目标，我们将重点做好以下工作：

（一）聚焦提质升级，全力做强工业经济。以打造传统产业和新动能两大高地为抓手，持续推动工业转型升级、提质提效和集聚发展，全力争当工业强省战略的排头兵。

加快主导产业转型步伐。出台落实工业高质量发展若干意见，大力实施传统产业改造提升、新兴产业培育、龙头培育带动、集群发展提升等四大工程，加快构建新型产业体系。深入落实支持新钢跨越发展30条措施，全力支持新钢向千亿级钢企迈进；出台《新余市用钢产业发展规划》，推动新钢实施一批本地化合作项目，不断延伸钢铁深加工产业链条。积极融入全省“2+6+N”产业跨越式发展计划，不断壮大新兴产业规模，加快落实支持锂电产业发展30条措施，力促锂电、光电信息和装备制造产业主营业务收入分别增长15%、12%和9%。积极探索打造安全产业、环保产业，大力发展智能制造，加快高新区数控机床产业园建设；深入实施“机器换人”计划，引导企业实施技术改造和装备升级，力争全年技改项目投资增幅达30%以上。出台支持电力发展30条，为工业发展提供更有力的能源保障。

不断提升园区承载能力。围绕打造“升级版”园区，全力打好园区功能完善提升战役，加快推进电镀产业园等公共服务平台建设，充分发挥已建成平台效益，持续推进高新区和其他园区基础设施建设，进一步增强园区承载能力。坚持亩产论英雄，努力提高有限资源要素的使用效率。全力开展园区集群式项目满园、扩园行动，加快处置“僵尸企业”，整合盘活园区闲置资源，引导企业积极向园区集聚。加快建筑科技产业园、装配式建筑产业园和华鼎集团香料产业园等特色产业园建设，鼓励引导县区因地制宜打造一批产业特色鲜明、发展前景广阔的“园中园”。

大力扶持民营经济发展。深入推进降成本优环境专项行动，全面落实各项惠企政策，大力开展中小企业提升专项行动，推动资源向优质企业聚集，培育省级以上“专精特新”企业10家、“小巨人”企业3家，确保新增入规工业企业38家，加快形成既有顶天立地的大企业，也有铺天盖地的小企业的生动发展格局。扎实开展企业精准帮扶，继续打造好“仙女湖夜话”政企交流品牌，帮助民营经济解决发展中的困难。进一步规范涉企执法行为，放宽民营企业准入领域，为民营经济营造更好的发展环境、更公平的竞争环境。

（二）强化项目建设，持续扩大有效投资。牢固树立“不抓项目是失职、不会抓项目是不称职”的导向，更加注重项目质量，千方百计引项目，狠抓落地建项目，以更多大项目、好项目支撑新余经济社会高质量发展。

推进重大项目建设。安排大中型建设项目165个，总投资达1004.6亿元。全力推进大唐新余二期异地扩建、新钢公司研发基地和生活服务基地向仙女湖区转移项目开工建设，按计划推进国电投分宜电厂扩建项目一台机组投入运行，力争蒙华铁路（新余段）建成通车，完成新余煤储基地和环城路主体工程，全面启动环城路连接线工程建设。切实抓好新钢百亿产业转型升级改造工程、赣锋锂业5万吨电池锂盐、北京升华15000台电梯等一批工业项目建设，助力工业转型升级。大力推进基础设施补短板，认真开展大岗山水库前期工作，加快江口水库提高运行水位的论证，从根本上解决全市生活、生产、生态用水安全问题；加快推进分宜通用机场建设，全力推动赣江货运码头、高铁站至分宜和上高快速通道等项目开工建设，加快构建覆盖渝钤大地的立体化综合交通运输网络。健全重点项目协调机制，更加注重项目调度，继续推进市领导挂点、市直单位帮扶和分季度、分县区集中开工重大项目机制，全面推行重点项目数字化、信息化管理，力争项目管理有新突破。

提升招商引资实效。牢固树立招商引资首位战略，强化产业链精准招商、配套招商，认真组织招商推介活动，争取在招大引强上有新突破。继续开展以商招商，探索建立“实体企业＋专业化的服务外包企业＋政府”的新型招商模式，充分发挥商会招商、乡贤招商平台作用，深入推进“渝郎回乡”工程，吸引更多“渝郎”返乡创业。全面落实《新余市促进经济发展办法》，及时兑现各类招商优惠政策，畅通企业诉求渠道，让企业在新余放心投资、安心发展。

拓宽融资筹资渠道。全面落实《防控金融风险支持实体经济发展的若干意见》，加快修复金融生态，撬动更多信贷资源支持实体经济发展。深化融资担保机制改革，推动市国信担保公司与银行构建可持续银担商业合作模式。继续发挥“财园信贷通”“财政惠农信贷通”“科贷通”等作用，有效缓解民营企业融资难题。全面落实企业上市“三年行动”计划，引导更多优质企业借助资本市场力量做优做强，力争亿铂电子、天欣源在港交所上市。精准研判政策走向，完善“三争”工作机制，全力做好项目储备和前期工作，力争争取上级资金增长10%。

（三）推进转型提速，加快发展第三产业。坚持把促进服务业发展作为拉动经济增长的突破口，紧盯新热点、开发新需求、培育新业态、创造新供给，加快推动服务业转型发展。

提档升级旅游产业。以旅游“双创”为抓手，出台支持旅游发展30条，大力实施乡村旅游提升工程，加快推进七夕小镇、宽心小镇、分宜麻纺小镇、客天下·百丈峰文旅小镇等一批重点项目建设，推动多产业融合发展和旅游业态升级，力争全域旅游示范区创建顺利通过国家验收；加大仙女湖景区旅游资源整合力度，力争央视七夕晚会继续在仙女湖录制，进一步唱响仙女湖“七夕”品牌，让美丽风光加快演绎成“美丽经济”。

优化壮大商贸流通。积极融入国家“一带一路”倡议，鼓励企业参与全省境外经贸园区建设，重点推进建筑业、农业、矿业在非洲和东南亚的投资开发合作。完成陆路口岸查验区一期建设，支持新余至宁波舟山港“五定”班列定期运行，努力把新余打造成赣西进出口物流中心和国际集装箱集散地。继续办好“天工杯”工业设计大赛、南方农业（工程）机械展销会和麻博会，不断提升展会影响力。全力打造智能制造高端论坛，着力提升新余高端制造、智能制造的影响力，吸引更多高端企业来新余发展。抓紧制定城区农贸市场建设和管理标准，加快解决农贸市场脏乱差的问题。

加快培育新兴业态。深入推进智慧新余建设，加快全市信息化资源整合步伐，推动数据资源共建共享。依托大数据产业基础，积极引进一流互联网企业，培育壮大本地互联网企业，推动云计算、大数据等新兴信息技术深度融入工业、教育、文化、医疗、餐饮、出行等领域。加快推动乡镇电商服务站提质升级，支持更多企业参与电商示范体系建设，促进电商和商贸物流协同发展。

（四）坚持解放思想，纵深推进改革创新。坚持用改革的办法、创新的举措、开放的胸襟破解发展难题，拓展发展空间，增强发展活力。

深化重点领域改革。全力推动新宜吉转型合作示范区建设，高质量编制好经济一体化发展规划，认真抓好第二批36个合作项目。深入推进开发区改革，推动管理制度和运营模式不断创新。稳妥有序抓好机构改革。积极推进市以下财政事权和支出责任划分改革。深入开展市级党政机关和市属国有企业国有资产盘活整合专项行动，确保国有资产保值增值。稳妥推进殡葬改革，完成51个农村公益性墓地、新余人文纪念园一期和分宜县殡仪馆一期工程建设。

发挥创新引领作用。全力争创国家创新型试点城市和省级军民融合发展创新示范区，深入实施加大全社会研发投入攻坚行动，加快构建以企业为主体、市场为导向、“产学研用金”相结合的技术创新体系，力争全社会研发经费投入占生产总值比重达1.65%。新增国家高新技术企业10家，申报省级以上科技项目50项。认真落实人才政策30条和支持新余学院发展30条措施，不断提高高校毕业生入余留余就业创业比例。充分发挥人才创新创造活力，力争每万人发明专利拥有量达3.25件。

全力优化政务环境。持续深化“放管服”改革，推动更多政务服务事项“一次不跑”或“只跑一次”，确保事项办理率达80%以上，加快实现所有事项网上办理，让数据多跑路、群众少跑腿。进一步优化并联审批流程，企业注册开办压缩至3个工作日。加快疏通企业和群众办事的“堵点”，全面推行“错时延时服务”，努力把新余建设成为全省“办事最方便”的城市。

（五）围绕精建细管，不断提升城市品质。深入践行“精心规划、精致建设、精细管理、精美呈现”理念，全面推进城市功能与品质提升三年行动，努力把新余建设成一座宜居宜业、精致精美、大气磅礴、形神兼备的城市。

坚持精心规划。统筹城市生产、生活、生态空间，充分考虑城市功能和市民需求，通过城市双修、城市设计，高起点、系统化编制城市风貌提升规划, 结合城市棚户区改造推进教育、医疗、养老、社区服务合理布局，以科学的规划描绘、框定城市整体美，推动城市建设上水平、有品位。

坚持精致建设。以生态修复、城市修补为依托，用好用足城区每一寸可利用、可开发、可建设的土地，加快实施一批彰显钢城特色、完善城市功能、提升城市品质的城建项目，着力提高城市综合承载能力。打通修建长青路北延段和奥林路，完成劳动北路、仰天岗西大道、阳光西大道、余新公路等部分路段改造或维修，全面提升道路通行能力；实施湿地公园环孔目江游步道工程，完成钟灵大道景观提升改造，做精城市美的空间；融合人文底蕴和城市风貌，打造赣西民俗特色街；完成丹江大桥防护设施改造、10处交通安全隐患点治理、31座城区公厕新建及改造，推进8条背街小巷、5个老旧小区、3个国有企业生活小区基础设施改造，改造新钢环山、燕子山等区域10367户棚户区，全面改善群众生产生活环境。

坚持精细管理。以管理创新、文明创建为抓手，扎实开展治脏、治乱、治堵、功能修补、生态修复、特色彰显、亮化美化、治理创新等八大专项行动，大力整治脏、乱、堵等“城市病”，下决心解决好市容市貌、占道经营、交通秩序混乱、架空线“蜘蛛网”、黑臭水体等突出问题，全力树立城市新风貌、凸显城市高品质、塑造城市好形象、展现城市新魅力。加快《城市管理条例》立法进程，促进城市秩序步入规范化、精细化、长效化管理轨道。充分发挥升级版“12345”政府服务热线作用，让广大市民参与城市同管共创。修订《物业管理办法》，多渠道改善无物业小区环境，全方位强化物业服务企业监管，着力解决物业服务标准不一、水平不高、物业费难收等难题。

（六）做实乡村振兴，着力激发乡村活力。全面落实乡村振兴规划，着力构建特色鲜明的优势产业体系、山清水秀的人居环境体系和文明和谐的乡村生活体系。

大力发展现代农业。全面落实“藏粮于地、藏粮于技”战略，按照“精、优、特”产业发展思路，建成4.6万亩高标准农田，积极引导农村承包地集中连片流转，加快推进现代粮食产业园、中田双孢菇和分宜双胞胎生态化养殖等一批重点项目建设，以农产品上行为主体发展农村电子商务，推动一产接二连三发展。加大农业关键技术攻关力度，力争粮油优质品率提升30%以上。深入实施绿色生态农业“十大行动”，重点扶持果业、油茶、中药材等特色产业发展，加快形成绿色发展、质量高端、品牌突出的农业特色产业发展新格局。纵深推进农村集体产权制度改革，大力开展6个省级发展集体经济试点村的试点工作，让农民持续增收致富。

全力建设美丽乡村。以完善功能、提升水平为着力点，纵深推进新农村建设，因地制宜打造一批乡村旅游型、绿色生态型、现代农业型、特色文化型、传统村落型的示范样板，让美丽乡村看得见山、望得见水、记得住乡愁。加大古村落保护力度，开展237座古桥保护行动，让农村留住乡愁古韵。深入落实农村人居环境整治三年行动计划，持续推进城乡环境综合整治，巩固提升“拆三房、建三园”成果，新改建农村公厕1000座以上，不断提升村容村貌。大力开展“四好农村路”建设，着力打造10条最美乡村公路，完成县道升级改造22.1公里、窄道升级改造80公里，基本实现行政村通客车。加快推进城乡供水一体化工程，力争年底实现全市50户以上的自然村自来水通水率达90%以上，50户以下具备通水条件的自然村当期开户率达50%。

着力打造文明乡风。依托村级综合文化服务中心等场所，传播宣传社会主义核心价值观，弘扬时代新风，凝聚实现乡村振兴的强大精神力量。开展寻找最美乡村教师、医生、村官、家庭等评选表彰活动，发挥模范榜样的示范带动作用，弘扬真善美，涵养崇德向善的文明乡风。大力推进文化惠民工程，深入开展“三下乡”活动，不断丰富农民业余生活。充分发挥商会、新农村建设促进会等乡贤平台作用，全力培育新乡贤文化。大力倡导移风易俗，让农村社会更加文明和谐。

（七）狠抓生态环保，共同呵护绿水青山。坚定不移走生产发展、生活富裕、生态良好的绿色发展之路，打赢打好污染防治攻坚战，为后代留住青山绿水、蓝天白云。

推进环境综合治理。继续抓好环保督察发现问题整改，全力推进各项整改任务落实。巩固提升“保家行动”成效，加快推进园区污水管网建设，确保工业企业污水应纳尽纳。配合省级层面探索建立袁河流域生态补偿机制，加快实施流域截污治污体系建设等系列水污染治理工程、城区雨污分流项目和水系连通工程，建成村镇生活污水处理设施94座，全力改善水环境质量。持续开展“四尘”“三烟”“三气”综合治理，大力推进钢铁、水泥、化工、建筑等重点行业领域环保监管，推动新钢煤气综合利用高效发电二期、分宜海螺环保节能升级技术改造等一批环保项目建成投用，持续开展非煤矿山综合整治，巩固主城区烟花爆竹禁燃禁放成效，确保空气质量优良率、PM2.5等约束性指标控制在省政府下达的目标范围内。强化固体废物堆存场所整治，加快土壤重金属污染修复，推动建筑垃圾、生活垃圾、餐厨废弃物、工业固废等废弃物资源化利用和无害化处理。

完善生态保护机制。严格实施生态红线管控，认真编制自然资源资产负债表，继续推进领导干部自然资源资产离任审计。全面建立市县乡村四级林长制体系，新增造林1.55万亩，打造更多的天然“氧吧”。加强行政执法和刑事司法衔接，严厉查处环境违法行为，切实把污染防治纳入法制化轨道。严格落实河长制湖长制，以河湖长制促进河湖长治。

着力发展绿色产业。支持建筑节能新技术新产品应用，大力发展装配式建筑；积极推行环境污染第三方治理模式，加快推动与中节能、中国林科院等企业和科研院所合作事项的落实，大力发展环保技术、环保设备和环保服务等“零污染”的新兴产业，努力走出生态保护和经济发展的双赢之路。

（八）坚持为民谋利，倾力增进民生福祉。践行以人民为中心的发展思想，着力保障群众基本生活，持续提升公共服务水平，努力办好50件民生实事，不断满足人民日益增长的美好生活需要。

扎实开展脱贫攻坚。全面贯彻落实打赢脱贫攻坚战三年行动部署，按照“核心是精准、关键在落实、实现高质量、确保可持续”的工作思路，切实抓好贫困人口动态管理，纵深推进八大扶贫工程，重点抓好产业就业扶贫规范提升，确保5000人脱贫、8个省级贫困村摘帽。扎实推进城镇贫困群众脱贫解困工作，筑牢群众基本生活保障底线，确保全面建成小康社会路上一个都不少。

实施就业优先战略。确保新增城镇就业2万人，新增转移农村劳动力1.4万人，城镇登记失业率控制在4.5%以内。加大创业贷款发放力度，力争发放创业担保贷款3.7亿元以上。

完善社会保障体系。深化养老保险制度改革，推动社会保险扩面提质。加大被征地农民参保力度，确保存量被征地农民纳入基本养老保险参保率达95%以上。健全各项救助保障制度，完善城乡社会救助体系。进一步提高“颐养之家”服务管理水平，加快居家和社区养老服务站点建设，引导民办养老机构规范发展，不断满足城乡居民的养老服务需求。

繁荣发展社会事业。大力推动虎跃学校、迎嘉中学、仙女湖一小等11所中小学新建，新钢中学、市五中、市逸夫小学等9所中小学扩建，加快建立教师编制动态管理机制，力争通过2-3年的努力，彻底解决困扰群众多年的城区中小学“大校额、大班额”难题。落实城镇小区配套幼儿园要求，有效扩大学前教育普惠资源供给。深化产教融合、校企合作，着力提升职业教育服务地方经济发展能力。不断巩固公立医院综合改革成果，大力实施家庭医生签约服务提升工程，努力将这项工作打造成全国样板；持续深化医保、医药、医疗“三医联动”改革，完善“分级诊疗”，扎实做好重大疾病防治工作，力争完成市中医院门急诊楼、市妇幼保健院门诊保健楼、袁河医院综合大楼和住院大楼主体工程建设。大力推进城南全民健身中心、2个多功能运动场等公共体育设施建设，推进群众体育、竞技体育和体育产业融合发展。深入推进全国文明城市、国家卫生城市和国家食品安全示范城市“三城”同创。纵深推进扫黑除恶专项斗争，深入推进“互联网+社会治理”行动，做实社区网格化管理，加快构建全方位立体化的平安网。落实联合接访机制，依法规范信访秩序，加快构建良好信访生态。牢固树立安全发展理念，全面落实安全生产责任制，坚决遏制重特大事故发生。完善应急体系建设，提高对各类突发事件的预警预防和应急处置能力。关心支持妇儿、老龄事业，发展慈善和残疾人事业。加强国防后备力量和人民防空建设，推进“双拥”和优抚安置深层次发展。

三、全面加强政府自身建设

推进新余高质量跨越式发展，对政府自身建设提出了新的更高要求。我们将铸牢忠诚之魂、勇闯发展新路、担当时代使命、提升服务效能、锻造过硬本领，着力打造“五型”政府。

打造忠诚型政府。牢固树立“四个意识”，始终坚定“四个自信”，坚决做到“两个维护”，坚决贯彻执行中央、省委省政府和市委决策部署，确保政令畅通、令行禁止。始终牢记全心全意为人民服务的根本宗旨，我们吃着新余的米、喝着新余的水，就要忠于新余人民，努力造福这一方水土和百姓。

打造创新型政府。面对日益激烈的地域竞争，我们必须把创新摆在发展全局的核心位置，坚持思想再解放一些、步子再大一些，让创新成为破解难题的“利剑”。把创新的焦点放在为群众除忧解困上，对群众诉求，要想“怎么办才行”，而不是“简单说不行”，确实想尽一切办法“怎么都不行”的，也要向老百姓讲清楚。

打造担当型政府。牢固树立“说一千道一万，不如拿出自己的成绩单来看”的理念，坚持“干”字当头、“实”字为要，努力练就敢担当的宽肩膀和善成事的真本领，使出自己的最大力气，拿出自己的最高水平，推动每一项工作落实落地。坚持正向激励和反向问责相结合，完善容错纠错机制，让广大干部放下包袱、撸起袖子，一心一意谋发展，脚踏实地干事业。

打造服务型政府。坚持把服务作为政府的天职，努力推动政府角色从“管理者”向“服务者”转变。加快建立和完善多样化政企沟通机制，像尊重老师一样尊重企业老总，像尊重科学家一样尊重企业家，让同一条船上的“自己人”有地方说话，有困难能讲，有正当路径能办，全力构建“亲”“清”新型政商关系。切实强化服务意识，及时回应群众诉求。

打造过硬型政府。深入开展调查研究，切实提高谋发展的眼光、解难题的功夫、抓落实的水平。坚持“先立规矩后办事”，守纪律、讲规矩、一切按规矩办事。全面落实党风廉政建设主体责任和“一岗双责”，驰而不息纠反“四风”，全面塑造政府清正清廉清明的良好形象。认真落实“三重一大”报告和政府法律顾问制度，抓好重大行政决策的合法性审查，主动接受人大法律监督、政协民主监督和社会各界监督，认真办理人大议案、政协提案及其意见建议。

各位代表！征程万里风正劲，重任千钧再奋蹄。让我们紧密团结在以习近平同志为核心的党中央周围，在省委、省政府和市委的坚强领导下，锐意进取、埋头苦干，为打造“五个高地”、决胜全面建成小康社会、再铸新时代“工小美”新辉煌而不懈奋斗！
